# Intestinal obstruction due to a left paraduodenal hernia: a case report

**DOI:** 10.1186/1752-1947-7-272

**Published:** 2013-12-13

**Authors:** Sidi Mohammed Bouchentouf, Ferdaous Raissouni, Hakim El Kaoui, Ahmed Bounaim, Mohammed Jidal, Abdelmounaim Ait Ali, Aziz Zentar, Khalid Sair

**Affiliations:** 1 Surgical Department, Military Hospital, Mohammed V University, Avenue des FAR, Hay Riyad, Rabat, 10000, Morocco; 2 Department of Gastroenterology, Military Hospital, Mohammed V University, Rabat, Morocco; 3 Department of Radiology, Military Hospital, Mohammed V University, Rabat, Morocco

## Abstract

**Introduction:**

A left paraduodenal hernia is a rare congenital malrotational anomaly of the midgut that occurs in the paraduodenal fossa of Landzert to the left of the fourth duodenum. It is responsible for approximately 1% of small bowel obstructions.

**Case presentation:**

We report a case of left paraduodenal hernia combined with small bowel obstruction in a 47-year-old Mediterranean woman who had a history of recurrent abdominal pain. An abdominal computed tomography scan showed a saclike mass clustered in the left upper quadrant but failed to yield a clear diagnosis. We describe the surgical anatomy of this disease and the emergency surgical management together with a short review of the literature.

**Conclusions:**

Even though a left paraduodenal hernia is rare, it must be suspected in any upper intestinal occlusion. The high morbidity and mortality rate of complicated cases should motivate preventive treatment in case of incidental operative discovery.

## Introduction

Paraduodenal hernias are the most common form of internal hernias and are responsible of approximately 1% of small bowel obstructions [1].

They were first described in the nineteenth century under various names: left paraduodenal hernia (LPDH), Treitz retroperitoneal hernia, hernia of the fossa of Landzert, mesentericoparietal hernia of Longacre, and hernia into the descending mesocolon of Callander [2].

We describe a case of LPDH revealed by acute intestinal obstruction and explain its surgical management together with a brief review of the literature.

## Case presentation

**Figure 1:**
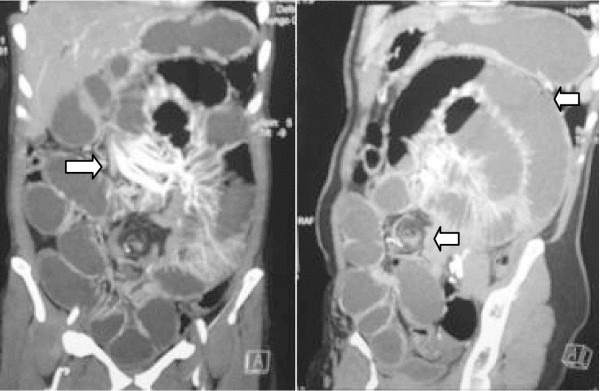
A computed tomography scan showing an aggregation of jejunal loops forming a retrocolic ‘pocket’ with a mass effect on the posterior stomach wall, a torsion zone at the root of the mesentery, and stretched and engorged mesenteric vessels.

**Figure 2:**
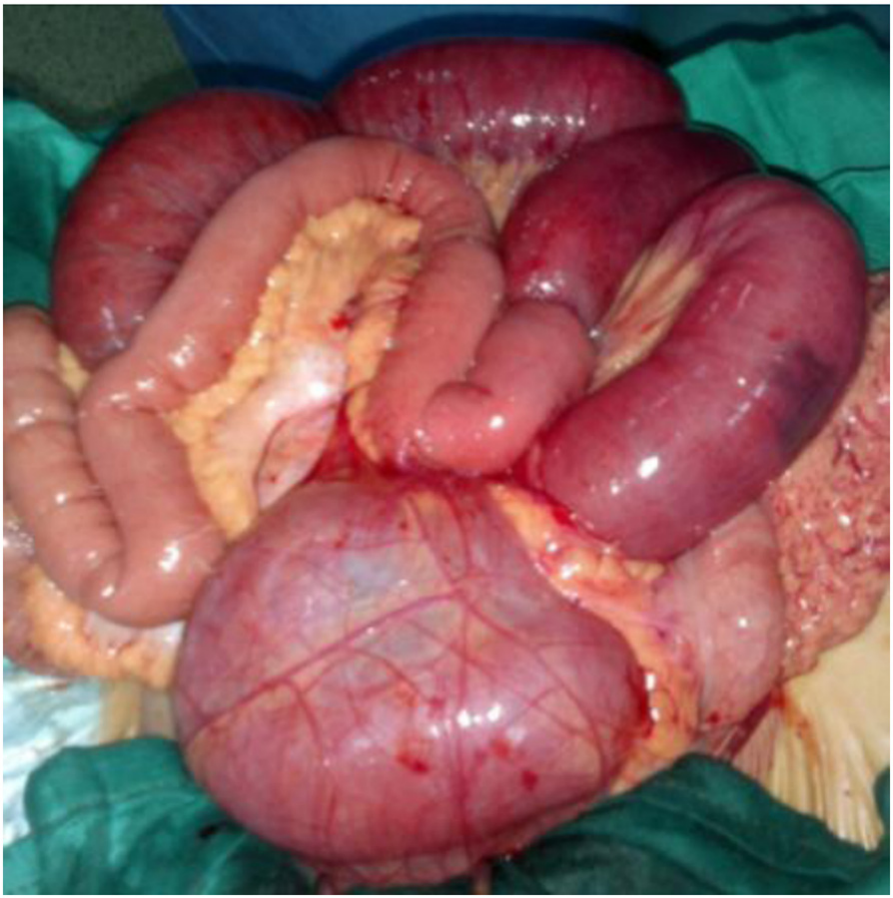
**Operative view.** Part of the jejunum was reduced, the other part is still in the retrocolic hernial bag and the level of strangulation is clearly visible.

A 47-year-old Mediterranean woman presented to our emergency department with abdominal pain and vomiting suggesting an acute small bowel obstruction. Her past medical history was marked by recurrent diffuse abdominal pain several years ago that resolved spontaneously or after taking phloroglucinol. An abdominal examination found abdominal distention with vaulting at the left hypochondrium. The umbilical, inguinal and crural area examination was normal. A computed tomography (CT) scan showed an aggregation of jejunal loops forming a retrocolic ‘pocket’ with a mass effect on the posterior stomach wall, a torsion zone at the root of the mesentery, and stretched and engorged mesenteric vessels (Figure 1). Intestinal volvulus or invagivation was suspected, however, the diagnosis was not assessed. The patient underwent emergent surgery. A median incision was performed, and exploration found a small bowel twisted upon its mesentery and entrapped in a large left paraduodenal space repressing forward the left mesocolon, the inferior mesenteric vein and the upper left colic pedicle. There was no intestinal necrosis (Figure 2). The bowel was reduced from the paraduodenal space, and then the volvulus was untwisted. The hernia sac was opened, respecting the vessels, and then the root of the mesentery was fixed to the posterior parietal peritoneum, with absorbable sutures closing the paraduodenal fossa. The patient was allowed to eat on the first day and left the hospital on the fourth day.

## Discussion

Paraduodenal hernias are the most common form of internal hernias and are responsible for approximately 1% of small bowel obstructions [1]. Found in 2% of autopsies [3], the LPDH occurs into the paraduodenal fossa of Landzert to the left of the fourth duodenum. In cases of hernia, this fossa is deep, forming a bag that may contain some or the entire small bowel, limited posteriorly by the posterior parietal peritoneum and edged in front by the inferior mesenteric vein and left superior colic vessels, which are stretched on the hernial bag.

This anatomical abnormality could be explained by two hypotheses [3]:

•The first one is the mechanical theory: increased abdominal pressure pushes the bowel in areas of low peritoneal adhesion;

•The second one seems more acceptable. It suggested that LPDH is secondary to errors in midgut rotation during the fifth to eleventh weeks of gestation, with the loops of bowel becoming interposed between the attachment of the mesentery and the posterior abdominal wall; the rotation of the midgut dorsally to the colic branches of the inferior mesenteric artery instead of ventrally allowing invagination into the mesocolon.

Even if it is a congenital disease, most of the cases are diagnosed between the fourth and sixth decades. The most common clinical presentation is intestinal obstruction [4]. Patients often report a history of vague abdominal pain, more often postprandially, varying with the change of position [1,3,4]. Occasionally, an abdominal mass may be palpable. Most patients remain asymptomatic, with LPDH incidentally discovered during laparotomy or autopsy [5]. The lifetime risk of incarceration of paraduodenal hernia (PDH) is reported to be approximately 50% and, as a result, it is recommended that all incidental PDH be surgically corrected.

A spiral CT scan appears to be the most helpful imaging for diagnosis; distended small bowel loops appear as encapsulated in a pouch that pushes the stomach forward; the mesenteric vessels look stretched and engorged [5-7].

In the case of occlusion, urgent surgery must be performed, the bowel must be reduced from the paraduodenal space and the hernia sac opened. Although the inferior mesenteric vein and the upper left colic vessels can be divided without compromising the blood supply of the colon, thanks to the shunt with the upper mesenteric system, they should be preserved whenever possible [8]. Intestinal resection is necessary in the case of strangulation and gangrene. The defect must be closed by fixing the root of the mesentery to the posterior parietal peritoneum with absorbable sutures. Some laparoscopic procedures have been reported as successful [5]. Mortality ranges from 20% to 50% for acute presentations, probably due to delayed diagnosis and vascular suffering of the proximal jejunum causing anastomotic leakage if resection and anastomosis are performed [9].

## Conclusions

A LPDH is rare but must be suspected in any upper intestinal occlusion. The high morbidity and mortality rate of complicated cases (for example strangulated hernia) should motivate preventive treatment in case of incidental operative discovery.

## Patient consent

Written informed consent was obtained from the patient for publication of this case report and any accompanying images. A copy of the written consent is available for review by the Editor-in-Chief of this journal.

## Abbreviations

CT scan: Computed tomography scan; (L)PDH: (Left) Paraduodenal hernia

## Competing interests

The authors declare that they have no competing interests.

## Authors’ contributions

SMB and FR wrote the manuscript. MJ prepared the iconography. All authors have read and approved the manuscript.
